# The relationship between outdoor sports atmosphere and study engagement among college students: the serial mediation of core self-evaluation and social-emotional competence

**DOI:** 10.3389/fpubh.2026.1965935

**Published:** 2026-10-23

**Authors:** Junhong Cui, Namei Song, Jie Ren

**Affiliations:** 1 Chongqing College of International Business and Economics, Chongqing, China; 2 School of Physical Education and Health Sciences, Chongqing Normal University, Chongqing, China

**Keywords:** college students, core self-evaluation, outdoor sports atmosphere, social-emotional competence, study engagement

## Abstract

**Objective:**

This study aims to explore the relationship between the outdoor sports atmosphere and study engagement among college students; examine the mediating and serial mediation effects of core self-evaluation and social–emotional competence on this relationship; and provide empirical evidence on how outdoor sports settings are associated with study engagement among college students.

**Methods:**

Using convenience sampling, 1,218 college students in Chongqing were selected as survey participants. They were administered the Outdoor Sports Atmosphere Scale, the Core Self-Evaluations Scale, the Social–Emotional Competence Scale, and the Utrecht Work Engagement Scale-Student. A structural equation model was constructed using AMOS 26.0, and the mediation effects were tested using the bias-corrected Bootstrap method (with 5,000 repeated samples).

**Results:**

The atmosphere of outdoor sports among college students was significantly and positively associated with study engagement (β = 0.223, *p* < 0.001). Core self-evaluation and social–emotional competence partially mediate this relationship, with a total indirect effect of 0.266, accounting for 54.40% of the total effect. All three mediating paths were significant: the indirect effect via core self-evaluation was 0.117, the indirect effect via social–emotional competence was 0.095, and the serial indirect effect via both was 0.054 (none of the 95% CIs included 0).

**Conclusion:**

The outdoor sports atmosphere on college campuses was not only directly and positively associated with study engagement but also indirectly associated with study engagement through three pathways: the mediating roles of core self-evaluation and social–emotional competence, and their serial mediation. Because the study was cross-sectional and study engagement was self-reported, the temporal or causal ordering of these associations cannot be established. These findings provide preliminary insights for future research on campus outdoor sports environments.

## 1 Introduction

Study engagement is a sustained, positive, and fulfilling psychological state associated with learning activities, with vitality, dedication, and focus as its core dimensions (1). Existing research indicates that study engagement not only positively predicts students’ academic performance (2) but is also closely associated with social adaptation (3) and life satisfaction (4). From a public health perspective, study engagement has been identified as a protective factor for mental health in previous research. Numerous studies have shown that academic engagement is closely linked to students’ positive emotions (5), levels of depression and anxiety (6), and mental health (7). It is also regarded as a key indicator for evaluating the quality of higher education and students’ personal development. In recent years, with the widespread penetration of digital media, college students’ attention has been increasingly diverted, leading to increasingly prominent issues such as insufficient motivation to learn, declining classroom participation, and a reduction in effective study time outside of class. Against this backdrop, how to effectively enhance college students’ levels of study engagement by optimizing the campus environment has become a central issue of shared concern among higher education practitioners and researchers.

The factors influencing college students’ study engagement are multidimensional. Previous studies have extensively explored the antecedents of study engagement from both the individual level [e.g., core self-evaluation, academic self-efficacy (8), and digital literacy (9)] and the environmental level [e.g., campus atmosphere (10), family background (11), teacher-student relationships (12), and teacher support (13)]—but few studies have incorporated both individual and environmental factors into a single integrated model to examine their synergistic effects on study engagement. It is worth noting that, as an integral component of physical education in higher education, the unique atmosphere fostered by outdoor sports has not yet received sufficient attention regarding its impact on college students’ psychology and behavior. From the perspective of ecosystem theory (14), the microsystems of individual development (such as the school and peer groups) exert a profound influence on an individual’s cognition, emotions, and behavior through daily interactions. As a key environmental factor within the campus microsystem, the outdoor sports atmosphere may, in theory, exert a positive spillover effect on students’ study engagement by shaping their self-concept and social–emotional competencies. However, environmental factors may be associated with behavioral outcomes not only directly but also indirectly through psychological, social, and emotional factors. This suggests that the relationship between the outdoor sports environment and study engagement may be mediated and serially mediated by core self-evaluation and social–emotional competence.

To provide an integrated theoretical foundation, this study synthesizes Ecological Systems Theory (14), Social Cognitive Theory (15), and Self-Determination Theory (16) into a unified “environment → cognition → emotion → behavior” framework. Ecological Systems Theory frames the outdoor sports atmosphere as a microsystem factor that influences students through daily environmental interactions. Social Cognitive Theory explains how such environmental input is internalized through cognitive appraisal—core self-evaluation—shaping positive self-perceptions. Self-Determination Theory further clarifies how social–emotional competence fulfills basic psychological needs, thereby being associated with motivated behavior. This integrated logic suggests the serial mediation pathway: outdoor sports atmosphere → core self-evaluation → social–emotional competence → study engagement. Based on this integrated theoretical framework, this study introduces core self-evaluation and social–emotional competence as mediating variables and constructs a serial mediation model to empirically address the following two core questions: First, does the outdoor sports atmosphere positively predict college students’ study engagement? Second, how do core self-evaluation and social–emotional competence function as mediators and serial mediators in this relationship? Specifically, this study contributes in three ways: (a) it examines perceived outdoor sports atmosphere (rather than physical activity behavior) as the starting point; (b) it identifies core self-evaluation and social–emotional competence as sequential mediators of study engagement; and (c) it integrates three theoretical perspectives into a unified framework. The study aims to provide new theoretical perspectives and empirical evidence for promoting college students’ study engagement from the perspective of campus sports environment development.

### 1.1 Outdoor sports atmosphere and study engagement

Grounded in the ecological component of our integrated framework, this section examines the direct relationship between outdoor sports atmosphere and study engagement. The term “atmosphere” is rooted in the concept of “cognitive maps” proposed by Tolman (17), which refers to an internal mental representation formed by individuals to understand their surroundings. In the field of education, classroom atmosphere (18) and school atmosphere (19) have been extensively studied and are regarded as important environmental variables influencing students’ academic achievement and psychological development. Building on this, this study defines the outdoor sports atmosphere as students’ perceived sports context—their overall perception of institutional support, peer norms, facility conditions, safety perceptions, parental encouragement, and structural constraints related to outdoor sports on campus. Importantly, this construct captures perceived environmental features rather than objective campus conditions or actual participation. In this sense, OSA reflects perception of the external environment, while core self-evaluation and social–emotional competencies reflect internal psychological resources, and study engagement reflects outcomes at the behavioral-motivational level. Compared to the atmosphere in traditional physical education classes, the outdoor physical activity atmosphere is characterized by greater openness, adventurousness, and social interaction; these unique attributes may be associated with distinct patterns in participants’ psychology and behavior.

Although research directly examining the relationship between the outdoor physical activity atmosphere and study engagement is limited, findings from related fields provide indirect support. Regarding the relationship between physical activity and positive psychological traits, a comparative study by Li et al. (20) found that outdoor education outperforms traditional physical education in enhancing college students’ resilience (including interest consistency and persistence). Research by Zhang and Li (21) further confirmed that a positive physical activity atmosphere significantly and positively predicts college students’ psychological resilience and growth mindset. These positive non-cognitive abilities (growth mindset, psychological resilience) have been shown to be closely related to study engagement (22, 23). Regarding the mental health benefits of outdoor activities, a systematic review by Down et al. noted that outdoor adventure education helps strengthen adolescents’ interpersonal connections and psychological strengths (24); a systematic review by Vasilaki et al. also found that outdoor activities such as hiking, cycling, camping, and nature exploration are closely associated with reduced anxiety and depression, improved mood, and enhanced environmental responsibility (25). These psychological benefits may help alleviate academic stress and have been associated with students’ academic adjustment. Furthermore, Zhang et al. found that pro-environmental behavior positively predicts college students’ study engagement (26); Slåtten et al. revealed that study engagement plays a role in the relationship between a supportive learning environment and college students’ academic performance (27). Xu et al. also found a significant positive correlation between the school atmosphere perceived by migrant children and their study engagement (10). The above evidence suggests that a positive campus environment and participation in physical activities may be indirectly associated with study engagement through individuals’ psychological resources, providing a theoretical foundation for this study’s exploration of the direct relationship between the outdoor sports atmosphere and study engagement.

### 1.2 The mediating role of the core self-evaluation

Aligned with the social cognitive component of our integrated framework, this section examines the mediating role of CSE between outdoor sports atmosphere and study engagement. Core Self-Evaluation (CSE) refers to an individual’s fundamental assessment of their self-worth, abilities, and sense of control, comprising four core traits: self-esteem, general self-efficacy, locus of control, and emotional stability (28). As a higher-order personality construct, CSE reflects an individual’s fundamental cognitive tendency regarding their ability to effectively control their environment and tasks, serving as a crucial cognitive link between the external environment and individual behavior.

Regarding the relationship between physical activity contexts and CSE, existing research has provided relatively consistent evidence. Physical exercise is significantly and positively correlated with CSE (29), and extracurricular physical activities can alleviate academic stress among college students by enhancing their CSE levels (30). A positive sports environment has also been shown to significantly and positively predict college students’ psychological resilience and growth mindset (21); psychological resilience and CSE overlap significantly in theoretical terms, as both involve an individual’s positive assessment of their own coping abilities. As an important contextual vehicle for physical exercise, an outdoor sports atmosphere may be associated with university students’ affirmative evaluations of their self-worth and abilities through the successful completion of challenging tasks, social comparison among peers, and positive feedback within teams. Regarding the relationship between CSE and study engagement, existing research consistently indicates a significant positive correlation between the two. Yan et al. (31) found that CSE was significantly positively correlated with all dimensions of study engagement, and that CSE directly predicted the vitality and focus dimensions of study engagement. Lei et al. (32) further confirmed that CSE mediates the relationship between professional identity and study engagement. These studies suggest that CSE, as a stable resource of self-perception, enables college students to face academic challenges with greater confidence and optimism, thereby making them more inclined to devote sustained energy and enthusiasm to their academic pursuits.

### 1.3 The mediating role of social–emotional competence

Corresponding to the self-determination component of our integrated framework, this section examines the mediating role of SEC between outdoor sports atmosphere and study engagement. Social–emotional competence refers to college students’ ability to build relationships with themselves, others, and the group, as well as their ability to make decisions that are responsible toward themselves, others, and the group (33). According to Self-Determination Theory (16), the development of SEC reflects the satisfaction of individuals’ basic psychological needs for competence, autonomy, and relatedness—needs that are essential for fostering intrinsic motivation and positive developmental outcomes.

Regarding the relationship between outdoor sports and SEC, although direct evidence is limited, existing research provides indirect support. Physical exercise significantly and positively predicts college students’ social–emotional competence (34); outdoor adventure education helps enhance adolescents’ interpersonal connections and psychological strengths (24); children’s time spent playing outdoors is significantly positively correlated with SEC (35). These findings suggest that characteristics inherent in outdoor activities—such as teamwork, emotional regulation, and social interaction—may be associated with the development of SEC among college students through the positive transfer of perceived atmosphere. Regarding the relationship between SEC and study engagement, existing research is relatively consistent. Qu et al. found that the social–emotional competencies of undergraduate students at research universities—such as open-mindedness, responsibility, and perseverance—have a significant positive impact on study engagement (36); Yang’s (37) study further confirmed that SEC positively predicts study engagement through indirect pathways involving teacher-student relationships, peer relationships, and school identification. A systematic review by Santos et al. (38) also indicates a positive correlation between study engagement and SEC. Furthermore, the responsible decision-making dimension within SEC helps college students better manage their relationships with peers, faculty, and the institutional community, thereby enhancing the effectiveness of study engagement (39). The above evidence suggests that the outdoor sports atmosphere may be indirectly associated with study engagement through a pathway, which involves shaping college students’ social-emotional competencies.

### 1.4 The serial mediation effect of core self-evaluation and social-emotional competence

Building upon the integrated theoretical framework outlined above—wherein ecosystem theory anchors the environmental input, social cognitive theory specifies the cognitive mediation, and self-determination theory accounts for the emotional -motivational mediation—this section further examines the serial mediation pathway. The theoretical rationale for the sequential order of core self-evaluation (CSE) before social–emotional competence (SEC) is grounded in the convergence of multiple theoretical perspectives. First, from the perspective of social cognitive theory (15), CSE represents a fundamental cognitive appraisal system that shapes how individuals interpret themselves and their environment. Individuals with higher CSE—characterized by elevated self-esteem, self-efficacy, and emotional stability—possess a more favorable cognitive foundation for engaging with social challenges and interpersonal interactions. This cognitive predisposition facilitates the acquisition and application of social–emotional skills. Previous research has found that self-efficacy and self-esteem play a serial mediating role in the effects of physical exercise on social–emotional competencies (40). Second, self-determination theory (16) provides a complementary lens: individuals with higher CSE are more likely to experience the satisfaction of their basic psychological needs for competence, autonomy, and relatedness—needs essential to the development of SEC. In this sense, CSE creates the internal conditions under which social–emotional competencies can flourish.

Empirically, individuals with high CSE, due to their higher self-esteem, self-efficacy, and emotional stability, are more likely to employ adaptive emotional regulation strategies such as cognitive reappraisal (41), exhibit lower negative emotional responses in interpersonal conflicts (42), and demonstrate higher levels of interpersonal adaptation (43). A workplace study by Yan et al. (44) confirmed that CSE is significantly and positively associated with emotional intelligence, and that emotional intelligence partially mediates the relationship between CSE and work-related well-being. Similarly, a study by Yao et al. (45) found that core self-evaluation and emotional intelligence serve as serial mediators in the relationship between online verbal aggression and interpersonal trust. Furthermore, emotional intelligence and SEC overlap significantly on the dimensions of emotional regulation and interpersonal relationships. Wang Guihong et al. (46) also found that CSE is significantly positively correlated with social adaptation ability. It is worth noting that a study by Fu et al. (47) found that social–emotional competence and CSE play a serial mediating role between social–emotional learning and school bullying. Although the sequence of variables in that study was “SEC → CSE,” this indirectly confirms their close association. Given that CSE is characterized by relative stability and a foundational nature, and in light of the integrative theoretical framework discussed earlier, we believe that stable self-evaluation provides the necessary cognitive and emotional resources for the subsequent development of social–emotional competencies.

The aforementioned theoretical analysis and empirical evidence collectively suggest a sequential association pattern of “environment → cognition → emotion → behavior”: the outdoor sports atmosphere may be associated with the development of social–emotional competence through core self-evaluation, and may further be associated with study engagement. Based on this, this study constructed a serial mediation model (see Figure 1) and proposed the following hypotheses:

**Figure 1:**
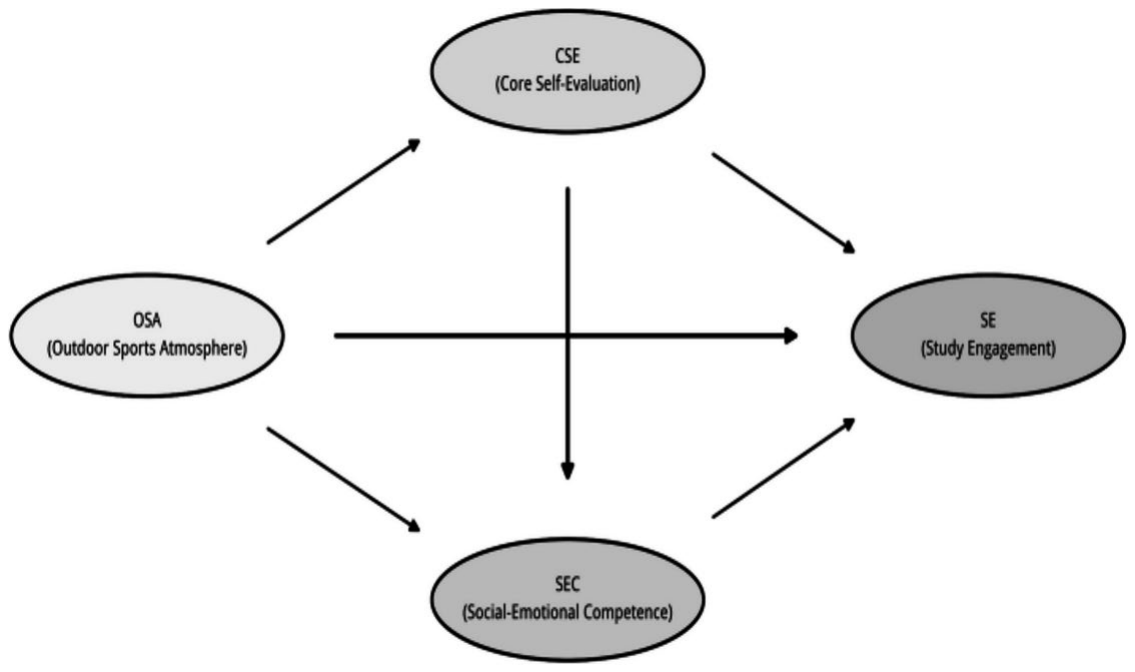
Hypothetical theory model.

*H1:* The outdoor sports atmosphere among college students positively predicts study engagement.

*H2:* Core self-evaluation mediates the relationship between the outdoor sports atmosphere and study engagement.

*H3:* Social–emotional competence mediates the relationship between the outdoor sports atmosphere and study engagement.

*H4:* Core self-evaluation and social–emotional competence exert a serial mediation effect on the relationship between the outdoor sports atmosphere and study engagement.

## 2 Materials and methods

### 2.1 Participants and research process

This study is a cross-sectional questionnaire survey, with data collection conducted from June to July 2026. Using convenience sampling, the study selected current college students (including undergraduate and postgraduate students) from four full-time general universities in Chongqing as the survey participants, covering a teacher-training institution (Chongqing Normal University), a comprehensive university (Southwest University), a science and engineering university (Chongqing University of Science and Technology), and a finance and economics university (Chongqing University of International Business and Economics). The research protocol was reviewed and approved by the Ethics Committee of Chongqing University of International Business and Economics (approval no.: XSWYH2026001), and all data collection and storage adhered to the ethical principles of the Declaration of Helsinki.

Before distributing the questionnaire, the required sample size was estimated *a priori* using PASS software. This estimation was based on a population proportion with specified precision, not a power analysis for structural equation modeling (SEM) or indirect effects. With a confidence level of 1 − α = 0.95 and a two-tailed confidence interval width of 0.06, the estimated minimum effective sample size was determined to be *N* = 1,098 (corresponding to a proportion confidence interval of [0.470, 0.530]). Taking into account an invalid questionnaire rate of approximately 20%, it was determined that no fewer than 1,373 questionnaires should be distributed, with the valid sample size required to be no less than 1,098. Furthermore, according to Kline, the minimum N:q ratio should be 10:1, which means that for each freely estimated parameter, at least 10 respondents are required (48). The final structural equation model in this study contains approximately 36 freely estimated parameters. This yields a minimum required sample size of approximately 360. Our final sample of 1,218 therefore exceeds this requirement. Participants were recruited online via the “Wenjuanxing” platform; survey links were posted by counselors at each school in the WeChat or QQ groups for the classes under their supervision. Students completed and submitted the questionnaires anonymously on a voluntary basis; a total of 1,400 questionnaires were distributed. Before completing the questionnaire, researchers explained to participants the purpose of the study, the anonymity of their responses, the voluntary nature of participation, and their right to withdraw from the study at any time without penalty. The system was configured to require participants to check the electronic option “I agree to participate” before accessing the main questionnaire; all participants signed an informed consent form online. All data were anonymized. During the data preprocessing stage, the exclusion criteria were as follows: (1) incomplete questionnaires (missing items ≥5%); (2) pattern-based responses (e.g., selecting the same option for more than 70% of the time); (3) failure of the consistency test for reverse-scored items; (4) completion time less than 1 min (less than one-third of the average completion time from the pilot test). Based on the above screening criteria, a total of 182 invalid questionnaires were excluded, resulting in a final sample of 1,218 valid responses (including 1,146 undergraduate students and 72 graduate students). The valid response rate was 87.0%, and the actual sample size exceeded the minimum requirement estimated in advance. The basic information for the final valid sample is as follows: In terms of gender distribution, there were 583 male students (47.9%) and 635 female students (52.1%); By academic year, there were 325 first-year undergraduates (26.7%), 320 second year undergraduates (26.3%), 296 third-year undergraduates (24.3%), 205 fourth-year undergraduates (16.8%), and 72 graduate students and above (5.9%); By major, 277 students (22.7%) were in the humanities, 456 (37.4%) in science and engineering, 228 (18.7%) in business and management, 195 (16.0%) in arts and sports, 41 (3.4%) in medicine, and 21 (1.7%) in other fields.

### 2.2 Measurement tools

#### 2.2.1 Outdoor sports atmosphere scale

The assessment was conducted using the Outdoor Sports Atmosphere Scale developed by Liu et al. (49). The scale consists of 17 items covering five dimensions: interpersonal connections, connection with nature, access to information, interpersonal barriers, and environmental barriers. It employs a 5-point Likert scale (1 = ”Strongly Disagree” to 5 = ”Strongly Agree”), with higher scores indicating a more positive perception of the outdoor sports atmosphere. Among these, six items—including “My parents do not encourage me to participate in outdoor sports,” “My school does not promote outdoor sports,” “None of my friends participate in outdoor sports,” “Although I’d like to participate in outdoor sports, I often cannot find a suitable venue,” “I feel that my safety is not guaranteed when participating in outdoor sports,” and “My academic workload is heavy, and I do not have time to participate in outdoor sports”—were reverse-scored items and were reversed prior to data analysis. In this study, the Cronbach’s α coefficient for this scale was 0.917.

#### 2.2.2 Core self-evaluations scale

The assessment was conducted using the Chinese version of the Core Self-Evaluation Scale, developed by Judge et al. and translated and revised by Du et al. (50). The scale consists of 10 items scored on a 5-point Likert scale (1 = ”Strongly Disagree” to 5 = ”Strongly Agree”). Among these, six items—”I often feel down,” “I doubt my abilities,” “I feel that many things are terrible and hopeless,” are reverse-scored items, and reverse-scoring was corrected prior to data analysis. Higher scores on all items indicate a higher level of core self-evaluation. In this study, the Cronbach’s α coefficient for this scale was 0.896.

#### 2.2.3 Social–emotional competence scale

The assessment was conducted using the College Students’ Social–Emotional Competence Scale developed by Chen et al. (33). This scale is based on the CASEL social–emotional learning framework and was localized to reflect the cultural characteristics of Chinese college students. It consists of 26 items covering four dimensions: self-relationship competence, other-relationship competence, group-relationship competence, and responsible decision-making competence. All items are positively scored using a 5-point Likert scale (1 = “Strongly Disagree” to 5 = “Strongly Agree”); a higher score indicates a higher level of social–emotional competence among college students. In this study, the Cronbach’s α coefficient for this scale was 0.936.

#### 2.2.4 Utrecht work engagement scale-student

The assessment was conducted using the Chinese version of the study engagement Scale developed by Schaufeli et al. and revised by Fang et al. (1). This scale was revised to reflect the cultural characteristics of the Chinese population and consists of 17 items covering three dimensions: vitality, dedication, and focus. All items are positively scored using a 5-point Likert scale (1 = “Strongly Disagree” to 5 = “Strongly Agree”); higher scores indicate a higher level of study engagement among college students. In this study, the Cronbach’s α coefficient for this scale was 0.927.

### 2.3 Data processing and analysis

This study used SPSS 26.0 and AMOS 26.0 for data management and analysis. First, the collected data were cleaned and organized, and then the Harman single-factor test was used to diagnose common method bias. Next, SPSS 26.0 was used to perform descriptive statistics, reliability analysis, and Pearson correlation analysis; AMOS 26.0 was used to conduct confirmatory factor analysis to test the scale’s construct validity and discriminant validity. Finally, based on the successful validation of reliability and validity, a structural equation model was constructed, and the mediation effects were tested using the bias-corrected percentile Bootstrap method (with 5,000 repetitions). During data collection, particular emphasis was placed on the anonymity and confidentiality of the questionnaires, and participants were explicitly informed that the data would be used solely for scientific research purposes to minimize common-method bias.

## 3 Results

### 3.1 Common method bias and measurement model

This study uses self-report questionnaires as data collection tools, with core variables assessed based on respondents’ self-ratings. This approach may give rise to common-method bias due to factors such as the single data source, scale item wording, and limitations in the study design. To avoid this systematic error, this study assesses the presence of common-method bias using Harman’s one-way analysis of variance. All questionnaire items were included in an exploratory factor analysis. The results of the unrotated exploratory factor analysis identified 13 factors with eigenvalues greater than 1. The maximum factor variance explained was 25.77%, which did not exceed the critical threshold of 40% (51). However, because Harman’s single-factor test has limited sensitivity and cannot demonstrate the absence of common-method bias, we further examined common-method variance using more robust approaches.

Specifically, we compared competing measurement models and conducted an unmeasured latent method construct (ULMC) analysis. As shown in the results in Table 1, the hypothesized four-factor model demonstrated good fit (χ^2^/df = 1.399, CFI = 0.996, RMSEA = 0.018) and fit substantially better than the three-factor, two-factor, and one-factor models. The one-factor model showed poor fit (CFI = 0.632, RMSEA = 0.161), and the ULMC model showed only marginal improvement over the four-factor model (ΔCFI = 0.002, ΔRMSEA = 0.004). These results suggest that common-method variance is unlikely to represent a major threat to the observed associations; nevertheless, because these procedures have limited sensitivity, it cannot be entirely ruled out.

**Table 1:** Comparison of competing measurement models.

Model	x^2^/df	GFI	AGFI	TLI	CFI	SRMR	RMSEA
4-factor	1.399	0.987	0.982	0.995	0.996	0.0192	0.018
3-factor	14.031	0.83	0.777	0.823	0.854	0.0866	0.103
2-factor	23.276	0.754	0.669	0.698	0.744	0.1097	0.135
1-factor	32.646	0.692	0.589	0.571	0.632	0.1085	0.161
ULMC	1.229	0.991	0.984	0.997	0.998	-	0.014

4-factor = four-factor model (OSA, CSE, SEC, SE as separate factors); 3-factor = three-factor model with CSE and SEC merged; 2-factor = two-factor model with OSA and SE merged and CSE and SEC merged; 1-factor = one-factor model with all items on a single factor; ULMC = unmeasured latent method construct model with a common method factor added to the four-factor model. ΔCFI and ΔRMSEA between 4-factor and ULMC = 0.002 and 0.004, respectively. SRMR was not available for the ULMC model.

To further establish discriminant validity among the four constructs, we conducted a joint confirmatory factor analysis (CFA) with all four latent variables included simultaneously. As shown in the results in Table 2, all constructs demonstrated adequate convergent validity (CR > 0.70; AVE > 0.50 for CSE, SEC, and SE; the AVE for OSA was 0.463, slightly below 0.50, but its CR was 0.812, and all factor loadings were significant, supporting convergent validity). For discriminant validity, the square root of the AVE for each construct exceeded its correlations with other constructs, satisfying the Fornell-Larcker criterion. Overall, these results support the distinctiveness of the four constructs and indicate that common-method bias is unlikely to have substantially influenced the findings.

**Table 2:** Convergent and discriminant validity.

Variables	CR	AVE	OSA	SEC	SE	CSE
OSA	0.812	0.463	**0.681**			
SEC	0.810	0.516	0.476	**0.719**		
SE	0.809	0.585	0.489	0.549	**0.765**	
CSE	0.901	0.752	0.465	0.513	0.516	**0.867**

The bold diagonal entries represent the square roots of the AVE, and the entries in the lower triangle represent the correlation coefficients between factors.

### 3.2 Descriptive statistics and correlation analysis

Before testing for mediating effects, this study conducted Pearson correlation analyses on each core variable to preliminarily examine the relationships among the variables. The results of the correlation analysis (see Table 3) show that the outdoor sports atmosphere is significantly positively correlated with core self-evaluation (r = 0.393, *p* < 0.001), significantly positively correlated with social–emotional competence (r = 0.385, *p* < 0.001), and significantly positively correlated with study engagement (r = 0.394, *p* < 0.001). Core self-evaluation was significantly positively correlated with social–emotional competence (r = 0.440, *p* < 0.001) and with study engagement (r = 0.439, *p* < 0.001). Social–emotional competence was significantly positively correlated with study engagement (r = 0.445, *p* < 0.001). The above results indicate that there are significant positive correlations between all pairs of the variables—outdoor sports atmosphere, core self-evaluation, social–emotional competence, and study engagement—providing a preliminary empirical basis for subsequent mediation analysis.

**Table 3:** Means, standard deviations, and correlation coefficients for each variable (*N* = 1,218).

Variables	M	SD	OSA	CSE	SEC	SE
OSA	3.356	0.761	1			
CSE	3.540	0.828	0.393***	1		
SEC	3.509	0.705	0.385***	0.440***	1	
SE	3.752	0.747	0.394***	0.439***	0.445***	1

*p < 0.05, **p < 0.01, and ***p < 0.001; the same applies throughout. OSA: Outdoor Sports Atmosphere; CSE: Core Self-Evaluation; SEC: Social–Emotional Competence; SE: Study Engagement.

### 3.3 Analysis of mediating effects

Based on the mediation effect testing procedure proposed by Wen et al. (52), this study constructed a structural equation model using AMOS 26.0 software and the Bootstrap method (with 5,000 repeated samples) to test the research hypotheses and path effects, setting a 95% confidence interval. First, the model fit was verified; the model fit indices are as follows: χ^2^/df = 1.399, GFI = 0.987, AGFI = 0.982, NFI = 0.985, RFI = 0.981, IFI = 0.996, TLI = 0.995, CFI = 0.996, SRMR = 0.019, and RMSEA = 0.018. These results indicate that the model has a good fit and is suitable for path analysis and mediation effect testing.

Next, we conducted a mediation analysis. The specific results are shown in see Table 4 and Figure 2, where Figure 2 displays the standardized path coefficients. The outdoor sports atmosphere was significantly and positively associated with on core self-evaluation (β = 0.465, *p* < 0.001); the outdoor sports atmosphere was significantly and positively associated with on social–emotional competence (β = 0.303, *p* < 0.001); core self-evaluation was significantly and positively associated with on social–emotional competence (β = 0.372, *p* < 0.001); the outdoor sports atmosphere was significantly and positively associated with on study engagement (β = 0.223, *p* < 0.001); core self-evaluation was significantly and positively associated with study engagement (β = 0.251, *p* < 0.001); social–emotional competence was significantly and positively associated with on study engagement (β = 0.314, *p* < 0.001). All of the above path coefficients reached statistical significance, and Hypothesis H1 is supported.

**Table 4:** Results of the mediator effect test.

Outcome variable	Predictive variable	β	B	SE	t	*p*	LLCI	ULCI
CSE	OSA	0.465	0.558	0.043	13.082	***	0.409	0.52
SEC	OSA	0.303	0.276	0.035	7.877	***	0.235	0.377
SEC	CSE	0.372	0.282	0.028	10.217	***	0.302	0.439
SE	SEC	0.314	0.351	0.045	7.736	***	0.234	0.39
SE	OSA	0.223	0.226	0.039	5.806	***	0.15	0.298
SE	CSE	0.251	0.212	0.031	6.793	***	0.177	0.319

β = standardized coefficient; B = unstandardized coefficient; SE = standard error of B. The t-values are computed as B/SE. LLCI/ULCI = lower/upper limits of the 95% confidence interval for the standardized coefficient (β). ***p < 0.001.

**Figure 2:**
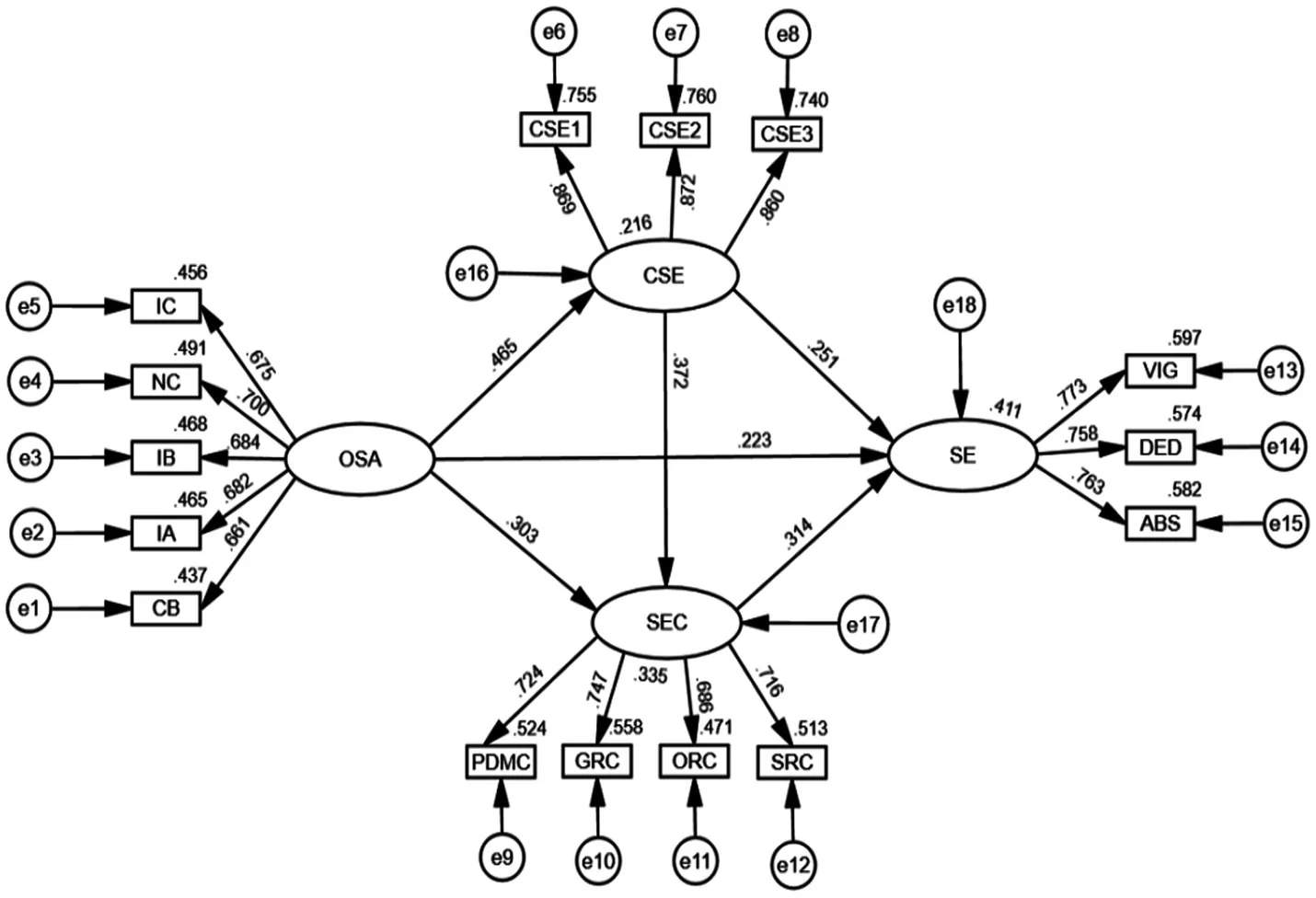
Path analysis diagram for standardized OSA, CSE, SEC, and SE. IC: Interpersonal Connection; NC: Nature Connection; IB: Interpersonal Barriers; IA: Information Access; CB: Conditional Barriers; SRC: Self-Relationship Competence; ORC: Other-Relationship Competence; GRC: Group-Relationship Competence; RDMC: Responsible Decision-Making Competence; VI: Vigor; DE: Dedication; AB: Absorption. In addition, since CSE is a unidimensional scale, the balanced method is used to bundle the observed indicators; CSE1, CSE2, and CSE3 represent the averages of the bundled indicators.

This study further used the Bootstrap method to test the significance of the mediating effect (see Table 5). The results show that the total indirect effect was 0.266 (BootSE = 0.023), with a 95% confidence interval of [0.224, 0.315] that does not include 0, indicating that the mediating effect is significant. The total effect was 0.489, and the direct effect was 0.223 (95% CI [0.150, 0.298]); the direct effect and the total indirect effect accounted for 45.60 and 54.40% of the total effect, respectively. The Bootstrap confidence intervals for all three specific mediation paths do not include 0, indicating that each mediation path is statistically significant: OSA → CSE → SE has an indirect effect of 0.117 (BootSE = 0.018), with a 95% CI of [0.083, 0.154], accounting for 23.93% of the total effect; The indirect effect of OSA → SEC → SE was 0.095 (BootSE = 0.017), with a 95% CI [0.067, 0.133], accounting for 19.43% of the total effect; The indirect effect of OSA → CSE → SEC → SE was 0.054 (BootSE = 0.009), with a 95% CI of [0.039, 0.075], accounting for 11.04% of the total effect. These results indicate that hypotheses H2, H3, and H4 are all supported. To further examine the robustness of the proposed mediation sequence, we also tested a reversed-order model (OSA → SEC → CSE → SE). As shown in Table 6, the reversed-order model yielded equivalent fit indices to the hypothesized model (ΔCFI = 0.000, ΔAIC = 0.000). This indicates that the cross-sectional data cannot determine the temporal precedence of CSE and SEC. The hypothesized order (CSE → SEC) is therefore retained on theoretical grounds, as elaborated in Section 1.4.

**Table 5:** Bootstrap test results for the mediating effect.

Effect types	Effect	Boot SE	Boot LLCI	Boot ULCI	Percentage of the total effect
Total effect	0.489	0.029	0.433	0.544	100%
Direct effect	0.223	0.038	0.150	0.298	45.60%
Total indirect effect	0.266	0.023	0.224	0.315	54.40%
OSA → CSE → SE	0.117	0.018	0.083	0.154	23.93%
OSA → SEC → SE	0.095	0.017	0.067	0.133	19.43%
OSA → CSE → SEC → SE	0.054	0.009	0.039	0.075	11.04%

Boot LLCI/Boot ULCI = lower/upper limits of the 95% confidence interval for the standardized coefficient.

**Table 6:** Comparison of alternative mediation models.

Model	x^2^/df	TLI	CFI	SRMR	RMSEA	AIC	BIC	ΔCFI	ΔAIC
Model A	1.399	0.995	0.996	0.0192	0.018	189.541	373.320	-	-
Model B	1.399	0.995	0.996	0.0192	0.018	189.541	373.320	0	0
Model C	2.678	0.977	0.982	0.0567	0.037	297.634	476.308	0.014	108.093

Model A = hypothesized serial mediation model (OSA→CSE→SEC→SE); Model B = reversed-order model (OSA→SEC→CSE→SE); Model C = parallel mediation model (OSA→CSE→SE; OSA→SEC→SE). ΔCFI and ΔAIC are relative to Model A.

## 4 Discussion

This study constructed and tested a serial mediation model to examine the relationship between the outdoor sports atmosphere for college students and their study engagement, as well as the underlying mechanisms. The results revealed that the outdoor sports atmosphere was significantly and positively associated with study engagement; core self-evaluation and social–emotional competence mediate this relationship independently, and together form a serial mediation pathway. These findings suggest that perceived environmental atmosphere may be associated with psychological resources, that social–emotional competence may precede study engagement, and that an integrated multi-theoretical framework can help explain this pathway. The following sections will provide a systematic discussion of the research findings.

### 4.1 The relationship between outdoor sports atmosphere and study engagement among college students

This study found that the outdoor sports atmosphere was significantly and positively associated with college students’ study engagement, and Hypothesis H1 was supported. This result can be explained from two perspectives: the ecological systems theory and self-determination theory.

From the perspective of the ecological systems theory (14), individual development is embedded within micro-, meso-, and macro-environmental systems. As the direct environment within the microsystem with which college students interact most frequently, the school’s atmospheric characteristics—including the outdoor sports atmosphere—are associated with psychology and behavior through activities, roles, and interpersonal relationships. The outdoor sports atmosphere is not merely a provision of sports opportunities but a composite context integrating exposure to nature, peer norms, autonomous participation, and low-competition social interaction; as a specific dimension of the campus microsystem, it provides students with channels for emotional recovery and stress buffering. A systematic review by Vasilaki et al. (25) indicates that activities such as hiking, cycling, outdoor camping, and nature exploration are closely associated with reduced anxiety and depression, improved mood, and enhanced environmental responsibility. This replenishment of emotional resources may provide the energy foundation for the “vitality” and “focus” dimensions of study engagement. From the perspective of self-determination theory (16), intrinsic motivation and engagement are sustained when the environment meets an individual’s needs for autonomy, competence, and relatedness. The peer norms, non-compulsory participation, and manageable challenges in natural settings characteristic of outdoor sports may be associated with opportunities to satisfy these three needs: autonomy (spontaneous participation rather than mandatory class time), competence (gradual mastery of routes and skills), and relatedness (acceptance within outdoor peer groups). The fulfillment of these needs may further be associated with the academic domain, potentially reflected in increased study engagement. Research by Yuan et al. indicates that school climate positively influences college students’ study engagement through the mediating effect of a sense of belonging to the school (53); Liu et al. confirmed that teachers’ academic and emotional support predict English study engagement through self-efficacy (54). This study narrows the focus of these findings from the general school atmosphere to the specific context of the outdoor sports atmosphere, thereby expanding our understanding of the environmental antecedents of study engagement. This also implies that campuses with a stronger outdoor sports atmosphere feature greater peer participation in sports and more active cultural promotion; may subtly be associated with students’ attitudes toward learning and their level of engagement. However, this study measured perceived sports context rather than actual participation. Therefore, the proposed mechanisms should be interpreted as theoretical possibilities rather than demonstrated pathways. Arey and Levental (55) found that sports participation was associated with positive self-perceptions and institutional affiliation among college students, regardless of the level of involvement. This further underscores the importance of distinguishing perceived sports context from actual participation in research on campus sports environments.

### 4.2 The mediating role of core self-evaluation

This study found that core self-evaluation mediates the relationship between the outdoor sports atmosphere and study engagement, thereby supporting Hypothesis H2. This finding provides empirical support for the core tenet of social cognitive theory—that “the environment influences behavior through cognitive evaluation”—while also revealing the synergistic effects of environmental factors and personality traits in the learning process.

As a positive environmental stimulus, an outdoor sports atmosphere may be associated with individuals’ positive perceptions of their own athletic abilities and self-worth through upward comparison and group identification effects when they perceive their peers actively participating in outdoor sports. Zhu et al. (30) have already demonstrated that extracurricular physical exercise alleviates academic stress among college students by enhancing their levels of core self-evaluation. This study expands the independent variable from “exercise behavior” to “sports atmosphere,” demonstrating that even if individuals have not yet frequently participated in sports, their self-evaluation system can still benefit as long as they perceive a supportive atmosphere. This aligns with the mechanisms of “observational learning and environmental suggestion” in social cognitive theory (15): a supportive atmosphere conveys positive signals, thereby enhancing individuals’ self-esteem, self-efficacy, and sense of control. Higher CSE is associated with greater study engagement, a phenomenon that can be understood through the relationship between personality traits and academic motivation. As a stable, dispositional personality resource, CSE has been shown to significantly predict college students’ study engagement (31); individuals with higher CSE typically exhibit greater emotional stability, higher self-efficacy, and more positive goal-oriented behavior, traits that help maintain sustained vitality, dedication, and focus during the learning process. Lei et al. (32) also found that CSE mediates the relationship between professional identity and study engagement. This study extends these findings to the context of outdoor sports, revealing an association pattern of “environmental perception → self-perception → academic behavior”.

### 4.3 The mediating role of social–emotional competence

This study found that social–emotional competence mediates the relationship between the outdoor sports atmosphere and study engagement, thereby confirming Hypothesis H3. This finding extends the research perspective on social–emotional competence from “the outcome of competence development” to “a precursor to study engagement,” thereby broadening the scope of SEC’s application in the field of educational psychology.

As a context that integrates exposure to nature, physical activity, and social interaction, the outdoor sports atmosphere provides a unique training ground for the development of SEC. In outdoor sports contexts, individuals may have opportunities to collaborate with peers, manage emotional experiences during physical activity, and cope with challenges and uncertainty—experiences that could potentially serve as practice for the various dimensions of SEC. Previous research has shown that experiences within learning communities can significantly and positively predict college students’ SEC (56). A more robust outdoor sports atmosphere may mean that students perceive more opportunities to participate in outdoor sports, engage in social interaction, and practice emotional regulation, thereby potentially promoting the development of SEC. The role of SEC in promoting study engagement can be understood from an “ability–motivation” perspective. Students with higher SEC are better at managing negative emotions (such as anxiety and frustration) during learning, are more adept at establishing positive relationships with teachers and peers, and are more inclined to adopt proactive coping strategies. These abilities may help them to cope more effectively with academic challenges, thereby maintaining a high level of study engagement. Previous research has found a significant positive correlation between SEC and study engagement (57); SEC can indirectly influence study engagement through a chain of mediating effects involving teacher-student relationships and a sense of belonging to the school (58). Building on this foundation, the present study tested these findings in the context of outdoor physical activity, suggesting an association pattern of ‘environment → emotion → behavior’ in which the outdoor sports atmosphere is associated with social–emotional competence and, in turn, with study engagement. However, actual participation was not measured in this study. Therefore, these proposed mechanisms should be interpreted as theoretical possibilities rather than demonstrated pathways.

### 4.4 The serial mediation effect of core self-evaluation and social–emotional competence

The core finding of this study is that core self-evaluation and social–emotional competence play a serial mediating role between the outdoor sports atmosphere and study engagement, thereby confirming Hypothesis H4. This result reveals a complete serial mediation pattern and provides a new theoretical perspective for understanding how environmental factors influence study engagement through multistage psychological processes.

This serial mediation pattern can be understood as involving three associated processes. The first process is ‘cognitive processes’: the outdoor sports atmosphere may provide opportunities for mastery experiences, social comparison, and positive feedback, which could potentially enhance an individual’s CSE. The second process is ‘emotional processes’: Higher CSE is associated with the development of SEC. Previous research has shown that CSE is significantly and positively correlated with interpersonal adaptation (43) and social adaptation (46), both of which are core domains of SEC. The third process is ‘behavioral outcomes’: higher SEC may be associated with individuals more effectively managing learning emotions, establishing positive interpersonal relationships, and coping with challenges in learning, thereby maintaining and enhancing their level of study engagement. This “environment → cognition → emotion → behavior” serial mediation pattern suggests the potential psychological process through which the outdoor sports atmosphere influences study engagement: from environmental perception to personality resources (CSE), to competence and literacy (SEC), and ultimately to learning behavior. This pattern reflects a multi-level association by which environmental factors influence study engagement more comprehensively than single-mediator models. At the same time, this serial mediation pattern indicates that CSE and SEC do not function in isolation but rather form a “cognitive–affective” continuum: CSE provides the cognitive foundation of “I can,” while SEC provides the competency assurance of “how I do it”; the two may work together in relation to study engagement. Existing serial mediation studies support this finding: Lei et al. (32) found that core self-evaluation and critical thinking play a chain-mediating role between professional identity and study engagement. This result corroborates the serial mediation findings of the present study, suggesting that study engagement is not directly associated with context or identity alone, but is associated with a two-stage psychological process from the “self-evaluation level” to the “social/cognitive competence level.” It should be noted that the reverse ordering (SEC → CSE) fit the data equally well, which is consistent with the cross-sectional design. The CSE → SEC sequence is theoretically imposed rather than empirically established. In summary, the serial mediation model constructed in this study provides a comprehensive association pattern—“environmental perception → cognitive resources → emotional competence → learning behavior”—for understanding the formation mechanism of college students’ study engagement. This study broadens the environmental perspective in research on student engagement; while these findings may provide preliminary guidance for institutions of higher education, their practical significance still needs to be validated through future longitudinal or experimental studies. Similarly, because actual participation was not measured, the mechanisms proposed in this stage—mastery experiences, social comparison, and positive feedback—should be regarded as theoretical possibilities rather than empirically established pathways. It should also be noted that study engagement was measured as a self-reported psychological state rather than an observed behavioral outcome.

### 4.5 Limitations and future directions

By constructing a serial mediation model, this study examined the relationship between the outdoor sports atmosphere on college campuses and students’ study engagement, as well as its underlying mechanisms, providing a new theoretical perspective for understanding how environmental factors are associated with learning behavior through multistage psychological processes. However, this study still has several limitations that require further refinement in future research. First, the cross-sectional design limits causal inferences. All variables in this study were collected at a single point in time, making it impossible to rigorously test the temporal relationships among them. Although the serial mediation model was constructed based on theory and the literature, cross-sectional data cannot provide direct empirical support for the temporal sequence among variables. Future research could adopt a longitudinal tracking design or an experimental intervention design to more rigorously test the causal pathways among variables. Second, there are limitations regarding sample representativeness and measurement methods. This study employed convenience sampling, with the sample drawn exclusively from four universities in Chongqing, which may affect the generalizability of the findings. Future research could be expanded to different geographic regions and institutional levels to test the model’s contextual stability. Additionally, all variables were measured via self-report. Specifically, study engagement was measured as a self-reported psychological state rather than an observed behavioral outcome. Although multiple procedures were used to examine common-method variance, these approaches have limited sensitivity, and common-method bias cannot be entirely ruled out; the results may still be affected by factors such as social expectations. Future research could incorporate peer evaluations, teacher evaluations, or multi-source data to enhance the robustness of the findings. Third, there is room for further development of the theoretical model. This study examined only two mediating variables—core self-evaluation and social–emotional competence. Future research could incorporate other psychological mechanisms (such as emotion regulation and self-efficacy) and include objective measures of gender, grade level, major, participation in sports, physical activity, and academic workload as control variables to further clarify the relevant boundary conditions and contextual factors, thereby enhancing the reliability and robustness of the research conclusions. Fourth, actual participation was not measured, so the proposed mechanisms (e.g., mastery experiences, teamwork, emotional regulation) remain theoretical possibilities. Future research should assess both perceived context and actual participation. No mental-health outcome was measured; therefore, claims regarding mental-health protection or promotion should be regarded as potential implications rather than demonstrated effects.

## 5 Conclusion

Based on survey data from 1,218 college students at four universities in Chongqing, this study constructed and tested a serial mediation model: “outdoor sports atmosphere → Core Self-Evaluation → Social–Emotional Competence → study engagement.” The study reached the following main conclusions: First, the outdoor sports atmosphere is significantly and positively associated with college students’ study engagement. Second, core self-evaluation mediates the relationship between the outdoor sports atmosphere and study engagement. Third, social–emotional competence mediates the relationship between the outdoor sports atmosphere and study engagement. Fourth, core self-evaluation and social–emotional competence act as serial mediators between the outdoor sports atmosphere and study engagement.

## Data Availability

The raw data supporting the conclusions of this article will be made available by the authors, without undue reservation.
